# Gut microbiota, behavior, and nutrition after type 1 diabetes diagnosis: A longitudinal study for supporting data in the metabolic control

**DOI:** 10.3389/fnut.2022.968068

**Published:** 2022-12-06

**Authors:** Deborah Traversi, Giacomo Scaioli, Ivana Rabbone, Giulia Carletto, Arianna Ferro, Elena Franchitti, Deborah Carrera, Silvia Savastio, Francesco Cadario, Roberta Siliquini, Franco Cerutti, Marilena Durazzo

**Affiliations:** ^1^Department of Public Health and Pediatrics, University of Turin, Torino, Italy; ^2^S.S.V.D. Endocrinology and Diabetology, O.I.R.M., Azienda Ospedaliera Città della Salute e della Scienza, Turin, Italy; ^3^Department of Health Science, Azienda Ospedaliero Universitaria Maggiore della Carità, University of Eastern Piedmont Amadeo Avogadro, Novara, Italy; ^4^S.C.U. Medicina Interna 3, Azienda Ospedaliera Città della Salute e della Scienza di Torino, Turin, Italy; ^5^Paediatric Endocrinology, Azienda Ospedaliero Universitaria Maggiore della Carità, Novara, Italy

**Keywords:** type 1 diabetes, microbiota, T1D progression, α-diversity, *Methanobrevibacter smithii*

## Abstract

**Introduction:**

Type 1 diabetes (T1D) risk involves genetic susceptibility but also epigenetics, environment, and behaviors. Appropriate metabolic control, especially quickly after the diagnosis, is crucial for the patient quality of life.

**Methods:**

This study aimed to produce a quantitative comparison of the behavior, nutrition habits, and gut microbiota composition between the onset and the 1-year follow-up in 35 children with T1D.

**Results and discussion:**

At follow-up, with the metabolic control, many parameters improved significantly, with respect to the onset, such as glycated hemoglobin (−19%), body mass index (BMI), and also nutritional behaviors, such as normal calorie intake (+6%), carbohydrate intake (−12%), extra portion request (−4%), and meals distribution during the day. Moreover, glycated hemoglobin decrement correlated with both total and rapid absorption carbohydrate intake (Spearman's rho = 0.288, 95% CI 0.066–0.510, *p* = 0.013), showing as the nutritional behavior supported the insulin therapy efficiency. The next-generation sequencing (NGS) analysis of microbiota revealed abundance differences for *Ruminococcus bromii* and *Prevotella copri* (higher at onset, *p* < 0.001) and the genera *Succinivibrio* and *Faecalibacterium* (lower at onset, *p* < 0.001), as a consequence of nutritional behavior, but it was not the only changing driver. The qRT-PCR analysis showed significant variations, in particular for Bacteroidetes and *Bifidobacterium* spp. (+1.56 log gene copies/g stool at follow-up, *p* < 0.001). During the year, in 11% of the patients, severe clinical episodes occurred (hypoglycemic or ketoacidosis). The likelihood of a severe hypoglycemic episode was modulated when the *Methanobrevibacter smithii* amount increased (odds ratio 3.7, 95% CI 1.2–11.4, *p* = 0.026). Integrated evaluation, including nutritional behavior and microbiota composition, could be considered predictive of the metabolic control management for children cohort with a recent diagnosis of T1D.

## Introduction

Diabetes includes a group of metabolic disorders characterized and identified by the presence of hyperglycemia in the absence of treatment. The heterogeneous pathological pathway includes defects in insulin secretion, insulin action, or both and disturbances of carbohydrate, fat, and protein metabolism. The long-term specific effects of diabetes include retinopathy, nephropathy, and neuropathy, among other complications (1–5).

Type 1 diabetes (T1D) makes up 5–10% of diabetes; in Europe, its incidence was 15 per 100,000 people and its prevalence was 12.2 per 10,000 people (6). Recent data showed a Thailand incidence of 17.6 per 100.000 people (7). In the T1D, β-cell-specific CD8 T cells destroy insulin-producing cells and consequently patients need insulin for survival (8, 9). The onset is most common in childhood and early adulthood, resulting in a relevant burden of disease in terms of healthy life year lost more than year file lost (6, 10). Strategies aimed at targeting stem-like autoimmune progenitor pools seem to emerge as novel and promising immunotherapeutic interventions (11).

The primary purpose of trials on T1D was, in general, treatment improvement, even if diagnosis and screening (3% of the studies) as health service and prevention (8% of the studies) were included (12). However, primary T1D prevention ways are not yet clear, even if genetic, social, and behavioral factors are relevant. From a biological point of view, in the last decade researchers focused their work on the elucidation of the role of the microbiota in T1D etiopathogenic process (13–15) and previous enteric virus infection (16). Gut microbiota dysbiosis was proposed as an inducing factor, strictly correlated with the education of host immunity during the early life (8). Coxsackievirus B (CVB) infection was proposed as a modulator of the β-cell autoimmunity, and a vaccine could be developed to reduce the T1D incidence (17, 18). Inactivated whole-virus vaccine covering all CVB serotypes (CVB1–6) was tested as safe and highly immunogenic in preclinical models including nonhuman primates (19) and also female NOD mice (16). Recent publications reported that SARS-CoV-2 accelerated the development of T1D. However, new cases of T1D, seemingly caused by COVID-19, are more likely due to earlier viral infections or other factors (5, 20).

Susceptible group identification can be based on screening, starting from genetic factors to genomics, including microbiome biomarkers (8, 21). A machine learning predictive model has been recently developed to predict those seroconverted patients not previously diagnosed. *Bacteroides spp*., in particular, *B. uniformis, B. dorei, and B. thetaiotaomicron*, decreased in the microbiota of patients with T1D, while *Prevotella copri* increased slightly and *B. vulgatus* was much higher (22). The most severe outcomes in patients with T1D are episodes of ketoacidosis that can bring to death in the absence of an effective treatment. Such severe conditions can be observed at the onset or after the diagnosis because of an inadequate metabolic control when some critical points are present, first a weak compliance with the glycemic self-control (13, 23). The crude ketoacidosis incidence rate, after diagnosis, was recently esteemed as 10.8% (7). Disease management, especially during the first period after the diagnosis, is crucial. A multifactor control strategy, in addition to the insulin therapy modulation, is needed (3, 24).

This study aimed to identify factors potentially related to weak metabolic control producing a qualitative–quantitative comparison of the behavioral, nutritional, and microbiota characteristics between the onset and the 1-year follow-up in a cohort of children with T1D.

## Materials and methods

### Study design and participants

The study began in January 2016, with the recruitment of patients at the onset (25), and ended in September 2018, after the conclusion of the follow-up for each included patient (follow-up phase of clinicaltrial.gov Protocol ID: G12114000080001). The work was conducted following the STROBE statement for a prospective study. The recruitment included 35 patients with pediatric diagnosed as affected by T1D in the two main pediatric hospitals in the Piedmont region as previously described (26). The inclusion criteria were recent diagnosis of T1D, age (5–10 years), normal weight, and residence in Piedmont. The exclusion criteria were celiac disease or chronic disease diagnosis, eating disorders, active infections, and residence changing. The included patients represent the most convenient possible sample (35 patients, of whom 12 were women) considering the onset cohort (40 patients). The sample size is small but not unusual in TD1 studies and represents the opportunistic cohort, collected during a full year of recruitment, including all the onsets with respect to the inclusion and exclusion criteria.

The guardians of the enlisting children read, understood, and then signed informed consent forms following the Declaration of Helsinki. A module is prepared for parents, children, and mature children (27). A questionnaire at the onset (26) and another similar questionnaire at the follow-up were given to the parents. Such a second questionnaire containing items and questions to retrieve data, especially to highlight changes, happened during the year on anthropometrics and on the family contest with particular regard to emotive stressors (such as mourning or separation) and socio-demographic, nutritional, and behavioral information. Moreover, at the follow-up, a new collection of biological samples (blood and stool) was performed, and additional glycated hemoglobin data were laboratory determined. The follow-up data on nutritional behavior (including updated nutritional anamnesis), anthropometrics, insulin needs, advanced technology adoption, additional therapies, and eventual hospital admission for ketoacidosis and hypoglycemic or hyperglycemic episodes were also collected by consultation of medical records. Nutritional intakes were collected using the 24-h dietary recall method. A face-to-face structured interview was conducted by a dietitian, who collected information with both children's and parent's help. Data concerning calorie and macronutrient supplies were estimated using the Food Composition database for Epidemiological Studies in Italy (28). Nutritional intakes were then compared with those recommended by LARN (Dietary Reference Intake for the Italian Population) and ADA (American Diabetes Association) (29, 30). Moreover, a questionnaire assessing general eating habits (such as snacking or extra portion practice) and specific food consumption (such as sweets) was also administered.

### Sample collection and DNA extraction

A kit for stool collection was delivered to each study participant following a validated procedure (31, 32) and using a Fecotainer device (Tag Hemi VOF, Netherlands). Fecal samples were homogenized within 24 h in the laboratory, and five 2-g aliquots were stored at −80°C until DNA was isolated. Total DNA from the stool samples was extracted using the QiaAmp PowerFecal DNA Kit (QIAGEN, Hilden, Germany). The nucleic acids were quantified using a NanoQuant Plate (TECAN Trading AG, Switzerland), which allows quantification using a spectrophotometer read at 260 nm. The spectrophotometer used was the TECAN Infinite 200 PRO, and the software was i-Control (version 1.11.10). The mean of the extracted DNA concentrations was 42.6 ± 32.4 ng/μL. The samples were stored at −20°C until molecular analysis was performed. All the oligonucleotides involved in the following biomolecular analysis are detailed in Supplementary Table 1.

### PCR-DGGE

The PCR products for denaturing gradient gel electrophoresis (DGGE) were obtained following the previously described method by amplifying the bacterial 16S rRNA genes following a marker gene analysis approach (33). All PCRs were performed with the T100 Bio-Rad Thermocycler. DGGE was carried out using a D-Code system (Bio-Rad) with a 30–50% denaturing gradient of formamide and urea (34). Electrophoresis was run at 200 V for 5 h at 60°C in 1X TAE buffer. Gels were stained for 30 min with SYBR Green I nucleic acid gel stain (10.000X in DMSO, S9430, Sigma-Aldrich, USA) and visualized using the D-Code XR apparatus from Bio-Rad. Then, DGGE bands were excised, incubated overnight at −20°C, washed, and crushed in 20 μl of molecular-grade water. The supernatant (2 μl) was used as a template and reamplified, as previously described, without BSA and using modified linker–PCR bacterial primers (357F-GC; 518R-AT-M13) (Supplementary Table 1) (35–43). The obtained PCR products were sequenced with Sanger sequencing (Genechron-Ylichron S.r.l.). The sequence similarities were obtained by the National Centre for Biotechnology Information (NCBI) database using the nucleotide basic local alignment search tool (BLASTn) analysis.

### NGS and qRT-PCR

High-throughput DNA sequencing and analysis were performed by BMR Genomics s.r.l. using the MiSeq 300PE Pro341F and Pro805R primer pair following the method previously described (26). The following microbial targets were quantified by qRT-PCR using a CFX Touch Real-Time PCR Detection System (Bio-Rad-Hercules, CA) and CFX Manager (3.1 Software): total bacteria, Bacteroidetes, *Bacteroides spp*., Firmicutes, *Bifidobacterium spp., Akkermansia muciniphila*, and *Methanobrevibacter smithii*, following the reaction condition and thermal protocol previously described (44). The PCR efficiencies were always between 90 and 110%. To confirm the amplification of each target, gel electrophoresis was performed on 2% agarose gels.

### Data elaboration and statistical analyses

The statistical analysis was performed using STATA version 16.0. A descriptive analysis of the variables was performed. Data were reported in absolute numbers and percent for categorical variables and means and standard deviations for continuous variables. Differences between the onset and follow-up were assessed by using Fisher's exact test categorical variables and Wilcoxon matched-pair signed-rank test for continuous variables. Univariable and multivariable linear regressions were then performed to estimate the impact of microbiota on the course of the disease (a level of glycated hemoglobin at follow-up, difference between the glycated hemoglobin at the onset and follow-up, and units of insulin administered daily). A *p*-value < 0.05 was considered significant for all analyses.

The DGGE gel analysis was performed with Bionumerics 7.2. The hierarchical classification was performed with a UPGMA system (1% tolerance and optimization level) and Pearson's correlation. Simpson's diversity index, Shannon's index, and Margalef's index were calculated for each DGGE profile to evaluate α-diversity.

The next-generation sequencing bioinformatics analysis was performed with the software pipeline Qiime2. The reads were cleaned up by the primers using the software Cutadapt (version 2018.8.0) and processed with DADA2, a package of the R software. The sequences were trimmed at the 3' end (forward: 270 bp; reverse 260 bp), filtered by quality, and merged with default values. Subsequently, the sequences were elaborated to obtain unique sequences. In this phase, the chimeras (denoised-paired) were also eliminated. The sequences were clustered against unique sequences at 99% similarity. The taxonomies of both GreenGenes (version 13-8) and Silva (version 132) were assigned to the OTU sequences. α-Diversity analyses were performed on all samples using the observed OTUs, Shannon, Pielou's evenness, and Faith PD indices, and for each index, the Kruskal–Wallis test was used to verify the significance of the comparisons between samples. β-Diversity analyses were performed on all samples using the Bray–Curtis, Jaccard, and UniFrac metrics (weighted and unweighted). Multivariable statistical analyses were performed using the PERMANOVA, Adonis, and ANOSIM tests; instead, the analysis of the differential abundance was based on the packages of R (MetagenomeSeq v 1.3.2, DeSeq2 v 3.15, and ANCOM-BC).

## Results and discussion

At the 1-year follow-up, the 35 patients injected a median of 17.5 units of insulin/day (IQR 7.25), equivalent to 0.54 units of insulin/day/pro Kg ± 37%, and 35.7% of them had more than four injections/day. Only one patient was equipped with an insulin pump. Such a project started a few years ago, while today, the proportion of the patients equipped with technologically advanced methods for the insulin treatment is reaching 100%. One-third of the cohort was yet in honeymoon (Table 1).

**Table 1:** Description of the clinically relevant data collected on the cohort at the 1-year follow-up.

		**Mean (±SD) or Number (%)**
Units of insulin /day/pro kg		0.54 (±0.2)
Honeymoon		11 (31.4%)
Insulin pump		1 (3.2%)
Insulin injection/day	3	1 (3.6%)
	4	17 (60.7%)
	>4	10 (35.7%)
Severe hypoglycemic episodes (number)	0	31 (88.6%)
	1	3 (8.6%)
	2	1 (2.8%)
Ketoacidosis		1 (2.9%)
Hospital admission due to hypoglycemic/hyperglycemic episodes ≥1		1 (2.9%)
Perceived life quality after the start of the insulin therapy	Better	8 (23.5%)
	Same	16 (47.1%)
	Worst	10 (29.4%)
Acceptance of the disease		27 (77.1%)

During the follow-up period, 12.9% of the patients had at least one severe hypoglycemic episode, while one ketoacidosis was observed. Such severe outcomes were all observed in patients with mothers with a low–medium education level (minor than high school) even if such result was not statistically significant (Fisher's exact = 0.096). The questionnaires' answers elaboration (see Materials and methods section) showed that only half of the cohort perceived quality of life changed by the insulin therapy, 23.5% in positive, while around 77% of the patients accepted the disease (Table 1).

Table 2 shows the main changes in the data collected at the follow-up with respect to the onset.

**Table 2:** Data collected from the questionnaire, nutritional anamnesis, and stool qRT-PCR microbial targeting analysis at the onset and 1-year follow-up.

		**Onset**	**Follow-up**	*p* ^*^
Glycated hemoglobin (%)		11.9 (2.1)	9.6 (11.0)	< 0.0001
**Nutritional anamnesis and behavior**
BMI (percentile)		37.9 (32.0)	60.8 (29.7)	< 0.0001
Total calorie intake (Kcal/die)		1957.1 (353.4)	1837.90 (308.3)	0.089
Nutritional relevance (Δ Kcal %)	Normal	19 (54.3%)	19 (61.3%)	0.006
	Deficiency	7 (20.0%)	11 (35.5%)	
	Excess	9 (25.7%)	1 (3.2%)	
Total supply of proteins (g)		67.0 (13.7)	69.4 (15.8)	0.464
Total Protein supply (pro kg)		2.4 (0.6)	2.1 (0.4)	0.0029
DELTA g/kg		1.4 (0.6)	1.1 (0.4)	0.0029
Total supply of lipids (g)		69.9 (14.2)	67.6 (14.4)	0.215
Total supply of lipids (%)		32.3 (4.8)	33.1 (3.6)	0.724
Total supply of carbohydrates (g)		266.1 (62.4)	237.1 (43.2)	0.0236
Total supply of carbohydrates (%)		54.1 (5.9)	51.7 (4.7)	0.1448
Nutritional relevance CHO %	Normal	30 (85.7%)	29 (93.5%)	0.071
	Deficiency	1 (2.9%)	2 (6.5%)	
	Excess	4 (11.4%)	0 (0.0%)	
Total supply of CHO RA (g)		85.7 (33.2)	64.9 (18.2)	0.0024
Total supply of CHO RA (%)		17.5 (5.6)	14.3 (3.9)	0.0110
Nutritional relevance CHO RA %	Normal	1 (2.9%)	5 (16.1%)	0.160
	Deficiency	9 (25.7%)	10 (32.3%)	
	Excess	25 (71.4%)	16 (51.6%)	
The child has access to food by himself when he/she is at home		21 (61.8%)	15 (48.4%)	0.339
The child consumes meals alone	Always alone	4 (11.8%)	3 (9.7%)	0.273
	Always with an adult	27 (79.4%)	27 (87.1%)	
	Both	3 (8.8%)	1 (3.2%)	
Number of extra meals a day	1	1 (2.9%)	4 (12.9%)	0.401
	2	12 (35.3%)	22 (71.0%)	
	3	13 (38.2%)	5 (16.1%)	
	4	8 (23.5%)	0 (0.0%)	
Does the child consume meals while watching TV?		20 (58.8%)	18 (61.3%)	0.098
Does the child consume sweets more than three times a week?		21 (61.8%)	6 (19.3%)	0.318
The child is happier to consume meals	At home	24 (70.6%)	23 (74.2%)	0.001
	At school	5 (14.7%)	6 (19.3%)	
	both	5 (14.7%)	2 (6.5%)	
Does your family consume meals all together?		28 (82.3%)	29 (93.5%)	0.747
Extra portion		20 (58.8%)	17 (54.8%)	0.013
Which one is the main meal of your child?	Lunch	7 (20.6%)	7 (22.6%)	0.016
	Dinner	25 (73.5%)	23 (74.2%)	
	Both	2 (5.9%)	1 (3.2%)	
Do you prefer to cook one-course meals or not?	One-course meals	6 (17.7%)	3 (9.7%)	0.005
	Not one-course meals	27 (79.4%)	27 (87.1%)	
	Both	1 (2.9%)	1 (3.2%)	
**qRT-PCR and** **α-diversity by DGGE**
*Akkermansia muciniphila°*		6.16 (1.28)	6.74 (1.44)	0.0111
*Bacteroides* spp.°		8.54 (0.99)	9.09 (0.61)	0.0066
Bacteroidetes°		7.72 (1.48)	9.14 (0.63)	< 0.0001
Total bacteria Probe°		9.39 (1.07)	9.94 (0.48)	0.0151
Total bacteria SYBR°		9.91 (0.71)	9.93 (0.47)	0.8146
Firmicutes°		10.97 (0.77)	10.04 (0.48)	< 0.0001
*Bifidobacterium* spp.°		6.16 (1.17)	7.72 (0.74)	< 0.0001
*Methanobrevibacter smithii 16S°*		5.37 81.34)	5.49 (1.46)	0.3750
*Methanobrevibacter smithii nifH°*		5.30 (1.20)	5.37 (1.35)	0.5156
Firmicutes/Bacteroidetes ratio		1.50 (0.45)	1.10 (0.06)	< 0.0001
Simpson's index		0.09 (0.03)	0.12 (0.04)	< 0.0001
Shannon's index		2.74 (0.29)	2.36 (0.28)	< 0.0001
Margalef's index		2.93 (0.77)	2.23 (0.43)	< 0.0001

The continuous variables were expressed as means and standard deviations; the categorical variables were expressed as absolute numbers and percentages. Adj p-value: adjusted for multiple comparisons.

* Fisher's exact test for categorical variables and Mann–Whitney U-test for continuous variables.

°(Log gene copies/g stool).

Concerning anthropometric data, an increase in mean BMI percentile has been observed: from 37.9 ± 32.0 kg/m^2^ at baseline to 60.8 ± 29.7 kg/m^2^ at follow-up. This growth was probably due to a physiological weight rebound, which was correlated with the correction of a previous hypo-insulinemic catabolic state (45). However, some authors have also attributed weight gain in children with new-onset T1DM to insulin therapy initiation, because of its anabolic properties (46).

The glycated hemoglobin decreased by 19%, while an improvement in the nutritional behavior of the cohort was observable.

As recommended by the International Guidelines, medical nutritional therapy was an integral part of T1D treatment. At diabetes onset, after nutritional habits assessment, a dietitian provided both general information concerning a healthy diet and specifics regarding diabetes (e.g., sugar limitation and fiber increase). A personalized diet plan was also provided to each child. The collected nutritional and behavioral data at the onset and follow-up were compared to optimal nutritional behavior as previously detailed (29, 30). In particular, although calorie intake was adequate in most of the children at baseline, sugar and protein intakes exceeded standard recommendations even if a different intake of sugar and protein in Piedmont children with respect to the other Italian children is not demonstrated (29). Other authors also reported this (47–49). Instead, 12 months after T1D onset, patients consumed less amounts of simple carbohydrates and a more adequate amount of proteins. Moreover, children asking for extra portions decreased, probably because of a more complete and satisfied diet. All these eating improvements can be considered as the result of a good nutritional therapy.

Of course, glycated reduction was mainly due to insulin therapy; however, some other correlations have also been observed for nutrients. As expected, a positive correlation has been observed between sugar and total carbohydrate intakes and glycated hemoglobin values (coefficient = 0.288; 95% CI 0.066–0.510, *p* = 0.013), while no relationship was noted for other macronutrients, confirming previous outcomes (50).

### Microbiota by DGGE and NGS

The DGGE analysis did not show a clear clusterization of the profile between patient samples at the onset with respect to the follow-up (Supplementary Figure 1); the difference between the branches was very limited (≈30 Pearson similarity index). The data on the single-cut bands showed the presence in the profile of *Bacteroides faecis, Enterococcus faecium*, and *Romboutsia timonensis* (more frequent in the onset) and *Urmitella timonensis* (more frequent in the follow-up) (Supplementary Table 2). However, such frequency can be considered only descriptive.

Considering the NGS results, from a global taxonomic point of view, the mean Firmicutes abundance was 44% at the onset, and it increased weakly at the follow-up (48%). The order *Aeromonadales* (*Succinivibrio genus*) was particular and increased, reaching 1.67% at the follow-up. In parallel, the *Enterobacteriales* decreased in the same group, 1 year after the diagnosis. Aggregating the rarefaction curves, a higher number of OUTs were observed in the follow-up with respect to the onset; however, such difference was not significant (Krustal–Wallis *p* = 0.115). The value distribution is very wide, and it is not possible to evaluate a significant difference. The change drivers could be multiple, including age and diet behavior; however, 1 year is a limited period to observe a substantial modification in children in the absence of a specific intervention.

**Figure 1:**
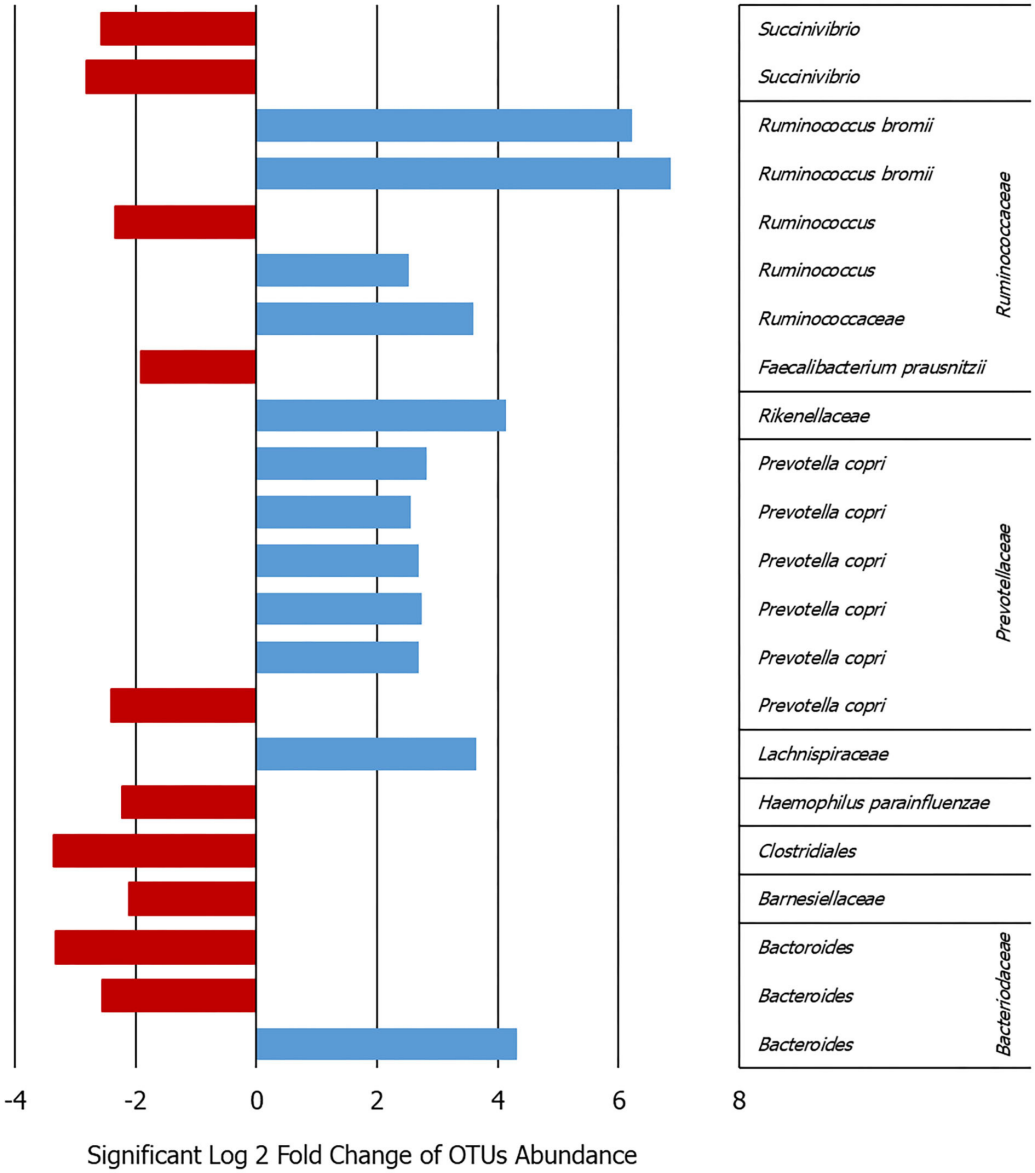
Bar graph showing the log2-fold change calculated by DeSeq2 in the patients between the onset and follow-up for the 22 OTUs that varied significantly in their abundance adj. *p* < 0.0009 (details given in Supplementary Figure 2).

Analyzing the OTU differential abundance, DeSeq2 showed 22 OTUs significant differences comparing the onset with follow-up abundances (*p* < 0.001). In detail, *Ruminococcus bromii* and *Prevotella copri* were higher bacteria abundance in the onset samples (adjusted *p* < 0.0001), while *Succinivibrio* and *Faecalibacterium* were significantly higher in the follow-up (adjusted *p* < 0.0001) (Figure 1 and Supplementary Figure 2). This evidence was confirmed by the analysis of the cDNA extracted from the same samples.

Some similar results can be deduced comparing the DGGE and NGS analyses in terms of major frequency or abundance in the samples between the onset and follow-up. In particular, for *Prevotella* not clear or opposite results can be observed for *Bacteroides, Fecalibaterium*, and *Succinivibrio* (Figure 1 and Supplementary Table 2). On the contrary, the DGGE can be considered, today, only a descriptive and low-sensitive method for such kind of analysis.

Previous data on comparable healthy children collected in the same area (26) showed several OTUs significantly higher in the healthy children with respect to the follow-up. In particular, *Bacteroides, Ruminococcus, Blautia*, and *Akkermansia* were higher in the healthy children with respect to the T1D follow-up microbiota (log2-fold change range from 2 to 6, adjusted *p* < 0.001). On the contrary, *Faecalibacterium* was higher in the T1D follow-up gut microbiota with respect to the healthy children.

During T1D metabolic control, the *Ruminococcaceae* family was enriched in patients with HbA1c < 53 mmol/mol (51). Moreover, a significantly higher relative abundance of *Ruminococcus* in T1D with respect to the healthy children with a lower relative abundance of *Bifidobacterium, Roseburia, Faecalibacterium*, and *Lachnospira* can be observed (52). Such data can induce a re-stabilization to eubiotic microbiota for the patients following the therapeutic management of the disease; however, a great variability of the results in terms of β-diversity can be observed in the literature, probably due to the population and individual variability. On the contrary, *Ruminococcus bromii* was a major taxon involved in the primary degradation of resistant starch, providing fermentation substrates and increased acetate concentrations for the growth of various major butyrate producers exhibiting a sulfite reducers and acetogens concurrently increased (53).

Recently, an abnormal expression of *Ruminococcus bromii* was observed also in the gut microbiota of gestational diabetes mellitus women (54), confirming a role of such microorganism in the diabetic diseases.

### qRT-PCR

In the comparison of the results for each microbial target, analyzed in the stool, at the onset and after 1 year, all the microbial targets were significantly modulated. The increase was, in general, below 1 log gene copies/g stool for all the targets, even if the most marked increase in both Bacteroidetes phylum and *Bifidobacterium* spp. (around +2 Log gene copies/g stool, *p* < 0.001) and a decrease in the Firmicutes phylum (−1 Log gene copies/g stool, *p* < 0.001) were observed (Table 2).

Bacteroidetes phylum and *Bacteroides* genus had an opposite development with respect to eubiosis reintegration as described in the literature. In particular, higher levels of various species of *Bacteroides* (e.g., *B. dorei*) were associated with the T1D development, and in our data, they continued such increment during the disease progression. On the contrary, Firmicutes and *Bifidobacterium* changed between the onset and follow-up, highlighting a restoration of gut microbial equilibrium.

Univariable linear regression model assessed an inverse correlation between glycated hemoglobin at follow-up and the delta between Shannon's index at the follow-up and onset (coefficient = −11.201; 95% CI −21.411; −0.992; *p* = 0.033). Such evidence suggests an increase in gut microbiota α-diversity was linked to lower glycated hemoglobin, collaborating for better metabolic control.

**Figure 2:**
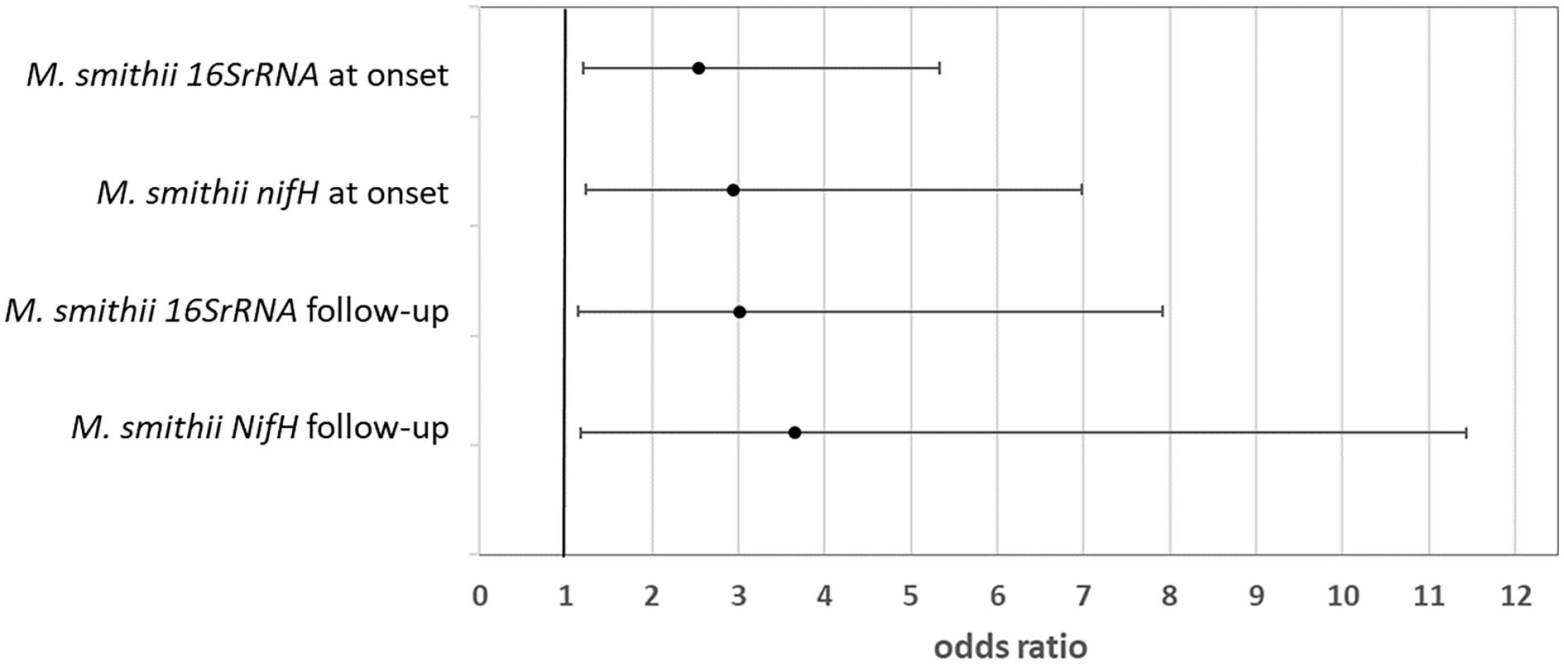
Likelihood of severe outcome after the diagnosis linked to a higher amount of *Methanobrevibacter smithii* in the patient stool at both the onset and follow-up (odds ratio and 95% CI).

Multivariable logistic regression model assessing the likelihood of severe hypoglycemic episode(s) in the last year based on the amount of *M. smithii* (log gene copies/g stool) at the onset and follow-up shows significant results (Figure 2). In particular, a higher colonization of the gut by *M. smithii* both at the onset and at the follow-up increased the risk of severe hypoglycemic episodes. Such severe outcome risk is slightly higher (up to four times with respect to low stool level of *M. smithii*) considering such methanogen presence at the follow-up. The association measures were adjusted for the percentile of BMI to avoid a confounding factor, and they showed a significant risk factor considering both the onset and follow-up *M. smithii* concentration and also the two target genes (*16S rRNA* and *nifH*) involved for the analysis of the same microbial target (*p* = 0.014, *p* = 0.025, *p* = 0.014, and *p* = 0.026). No behavioral or nutritional variables showed the modulation of such severe outcomes. The involvement of the methanogens in the dysbiosis of the intestinal microbiota was just widely confirmed in the literature (55). Such connection was mainly due to *M. smithii*, followed by *M. stadtmanae* and *M. luminyensis*. The methanogen level decreases during Crohn's disease, ulcerative colitis, and malnutrition, while it increases during diverticulosis, irritable bowel syndrome, colorectal cancer such as also in constipation and obesity. Despite the study limits, including the small sample size, to the best of our knowledge, the association between the higher level of *M. smithii* and severe outcomes during T1D progression is first shown.

*Methanobrevibacter smithii* produces methane that can contribute to slowed gastrointestinal motility, letting more time for energy harvest from the diet (56–58). This theory is the basis for the biological demonstration of the associations between *M. smithii* counts and weight gain. *M. smithii* allows for increased fermentation of carbohydrates to SCFAs that can provide energy to the host. In the few patients in which *M. smithii* was detected at a level > LOQ (23% of the included patients), such microbiota components seem to have a not treasurable role in the metabolic control probably following synergic interaction with other microorganisms such as the *Bacteroides*.

## Conclusion

Type 1 diabetes is a disease that needs a multidisciplinary approach both for the incidence reduction and for the prevention of the severe outcomes after the diagnosis. In association with insulin therapy, diet treatment is an integral part of diabetes management as it is able to influence glucose control, as also demonstrated in such a study. Moreover, the diet treatment was effective in such a T1D patient cohort showing significant modifications among the most diet behavior and parameters evaluated. Diet management plays an essential role, supporting the insulin treatment, in the prevention of diabetes complications through inflammation pathways modulation and cardiovascular risk reduction. The gut microbiota changes go with the disease progression and clinical and nutritional interventions, showing significant modulations between the onset and follow-up. The α-diversity as some microbial targets seems to be predictive of an adequate metabolic control and also of the likelihood of severe negative episodes due to a weak glycemic control. *Ruminococcus bromii* and *Bifidobacterium* spp. can be proposed as relevant microbial features for the gut eubiosis, while *M. smithii* can be considered as a proxy for severe outcomes after the onset, highlighting a higher-risk subgroup of patients that need the development of a dedicated disease management protocol.

## Data availability statement

The datasets presented in this study can be found in online repositories. The names of the repository/repositories and accession number(s) can be found below: https://www.ncbi.nlm.nih.gov/, PRJNA847791.

## Ethics statement

The studies involving human participants were reviewed and approved by Comitato etico interaziendale A.O.U. Città della Salute e della Scienza di Torino e Ordine Mauriziano di Torino ASLTO1, with record number 0117120 and Comitato etico Interaziendale A.O.U. Maggiore della Carità ASL BI, NO, VCO record number 631/CE, date: 11/25/2015. Written informed consent to participate in this study was provided by the participants' legal guardian/next of kin.

## Author contributions

FC, RS, and MD contributed to conceptualization. IR, DT, and FCa contributed to methodology. GS was involved in statistics. IR, DT, and SS contributed to validation. DT and GC performed the biomolecular analysis. GC, AF, and DC were involved in data collection and samplings. AF and DC performed the nutritional data analysis. FC contributed to resources and funding acquisition. AF, GC, and EF were involved in data curation. DT, GS, and AF were involved in writing—original draft preparation. DT and IR were involved in writing—review and editing. DT was involved in visualization. RS and MD were involved in supervision. RS, MD, and FCa were involved in project administration. All authors contributed to the article and approved the submitted version.

## Funding

This research was funded by the Italian Ministry of Health (RF-2011-02350617). Moreover, the University of the Study of Torino, the Città della salute e della scienza di Torino and the Hospital “Maggiore della Carità” di Novara co-funded such project. The publication fee was funded by the University of Torino.

## Conflict of interest

The authors declare that the research was conducted in the absence of any commercial or financial relationships that could be construed as a potential conflict of interest.

## Publisher's note

All claims expressed in this article are solely those of the authors and do not necessarily represent those of their affiliated organizations, or those of the publisher, the editors and the reviewers. Any product that may be evaluated in this article, or claim that may be made by its manufacturer, is not guaranteed or endorsed by the publisher.
