# Supplementary material for: Gut microbiota, behavior, and nutrition after type 1 diabetes diagnosis: A longitudinal study for supporting data in the metabolic control
Source: Front Nutr. 2022 Dec 6;9:968068. doi: 10.3389/fnut.2022.968068 (PMC9763620; doi:10.3389/fnut.2022.968068)
Supplement: Supplementary file 1 [file Data_Sheet_1.pdf]

Supplementary Material

Supplementary Table 1 – Details on the oligonucleotide sequence involved in the study for the biomolecular analysis and corresponding reference.

Microbial Target		Sequences	Standard genomico	Ref
Total Bacteria<br>16S DNA	Primer F<br>Primer R	5′ACTCCTACGGGAGGCAGCAG3'<br>5′ATTACCGCGGCTGCTGG3’	*Desulfovibrio vulgaris*<br>ATCC 29579D-5	(Harbison et al., 2019)
Total Bacteria<br>16S DNA	Primer F<br>Primer R<br>Probe	5′AGAGTTTGATCMTGGCTCAG3’<br>5′TTACCGCGGCKGCTGGCAC3’<br>5′CCAKACTCCTACGGGAGGCAGCAG3’	*Desulfovibrio vulgaris*<br>ATCC 29579D-5	(Harbison et al., 2019)
Bacteroidetes<br>16S rDNA	Primer F<br>Primer R	5′CATGTGGTTTAATTCGATGAT3'<br>5′AGCTGACGACAACCATGCAG3'	*Bacteroides fragilis*<br>ATCC 25285D-5	(Murri et al., 2013b; Maffeis et al., 2016)
Bacteroides 16S rDNA	Primer F<br>Primer R	5′GAGAGGAAGGTCCCCCAC3'<br>5′CGCTACTTGGCTGGTTCAG3'	*Bacteroides fragilis*<br>ATCC 25285D-5	(Murri et al., 2013b; Maffeis et al., 2016)
Firmicutes 16S rDNA	Primer F<br>Primer R	5′ATGTGGTTTAATTCGAAGCA3'<br>5′AGCTGACGACAACCATGCAC3'	*Clostridium acetobutylicum*<br>ATCC 824D-5	(Pellegrini et al., 2017)
Bifidobacteria<br>16S rDNA	Primer F<br>Primer R	5′CTCCTGGAAACGGGTGG3'<br>5′GGTGTTCTTCCCGATATCTACA3'	*Bifidobacterium longum infantis*<br>ATCC 15697-D	(Qi et al., 2016)
Akkermansia muciniphila 16S rDNA	Primer F<br>Primer R	5′CAGCACGTGAAGGTGGGGAC3'<br>5′CCTTGCGGTTGGCTTCAGAT3'	*Akkermansia muciniphila*<br>ATCC BAA835D-5	(Maffeis et al., 2016)
M. smithii 16S rDNA	Smit.16S-740 F<br>Smit.16S-862 R<br>Smit.16S FAM	5′CCGGGTATCTAATCCGGTTC-3’<br>5′CTCCCAGGGTAGAGGTGAAA3’<br>5′CCGTCAGAATCGTTCCAGTCAG3’	*M. smithii*<br>DSM 861	(Harbison et al., 2019)
M. smithii nifH	Mnif 202 F<br>Mnif 353R<br>Mnif Probe	5′GAAAGCGGAGGTCCTGAA3'<br>5′ACTGAAAAACCTCCGCAAAC3'<br>5′CCGGACGTGGTGTAACAGTAGCTA3'	*M. smithii*<br>DSM 861	(Stewart et al., 2018)
Bacterial<br>16S rRNA	357 F-GC<br>518 R	5′GCclampCTCCTACGGGAGGCAGCAG3'<br>5′GTATTACCGCGGCTGCTGG3'		(Mejía-León et al., 2014)
V3-V4<br>16S rDNA	Pro 341 F<br>Pro 805 R	5′TCGTCGGCAGCGTCAGATGTGTATAAGAGACAGCCTACGGGNBGCASCAG3'<br>5′GTCTCGTGGGCTCGGAGATGTGTATAAGAGCAGGACTACNVGGGTATCTAATCC3'		(Putignani et al., 2014)

Supplementary Figure 1 – Dendrogram of phylogenetic analysis of the DGGE bacteria profile. The Pearson similarity index was reported close to the dendrogram nodes.

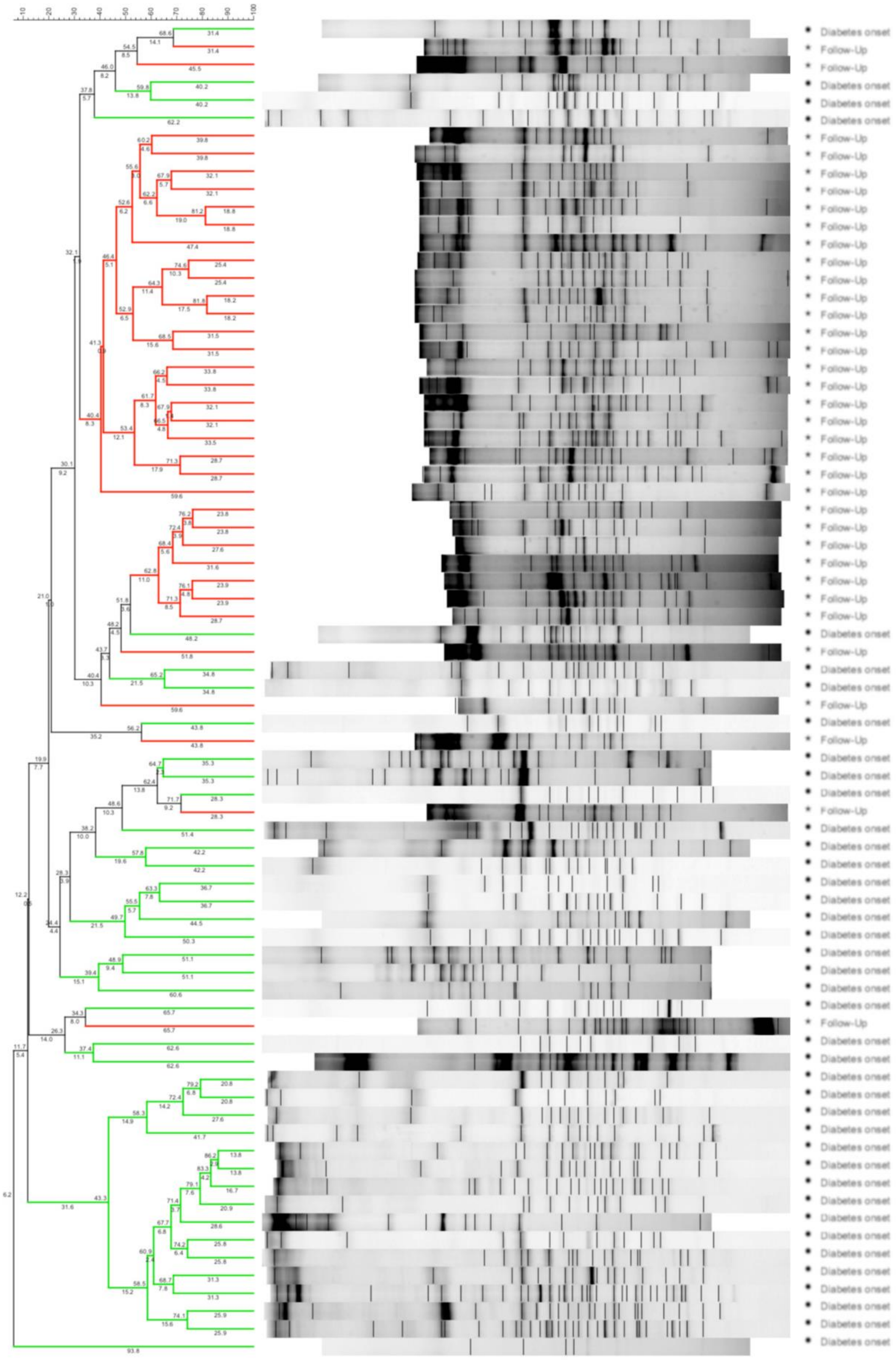

Supplementary Table 2 – Description and frequency of detection of the main bacteria species identified by the sequencing of DGGE bands in onset and follow-up samples. The last column showed the frequency as percentage on the total sample and number within the brackets.

Closet Relative	Identity	Phylum	Class	Order	Family	Genus	Onset	Follow-up
Alistipes timonensis	96%	Bacteroidetes	Bacteroidia	Bacteroidales	Rikenellaceae	Alistipes	75% (30)	60% (21)
Bacteroides coprocola	98%	Bacteroidetes	Bacteroidia	Bacteroidales	Bacteroidaceae	Bacteroides	5% (2)	0% (0)
Bacteroides dorei	100%	Bacteroidetes	Bacteroidia	Bacteroidales	Bacteroidaceae	Bacteroides	60% (24)	74.3% (26)
Bacteroides faecis	99%	Bacteroidetes	Bacteroidia	Bacteroidales	Bacteroidaceae	Bacteroides	37.5% (15)	11.4% (4)
Bacteroides finegoldii	94%	Bacteroidetes	Bacteroidia	Bacteroidales	Bacteroidaceae	Bacteroides	10% (4)	0% (0)
Bacteroides intestinalis	97%	Bacteroidetes	Bacteroidia	Bacteroidales	Bacteroidaceae	Bacteroides	92.5% (37)	91.4% (32)
Bacteroides timonensis	100%	Bacteroidetes	Bacteroidia	Bacteroidales	Bacteroidaceae	Bacteroides	7.5% (3)	0% (0)
Barnesiella intestinihominis	100%	Bacteroidetes	Bacteroidia	Bacteroidales	Barnesiellaceae	Barnesiella	57.5% (23)	68.6% (24)
Blautia faecis	99%	Firmicutes	Clostridia	Clostridiales	Lachnospiraceae	Blautia	82.5% (33)	91.4% (32)
Butyricimonas paravirosa	97%	Bacteroidetes	Bacteroidia	Bacteroidales	Odoribacteraceae	Butyricimonas	20% (8)	0% (0)
Clostridium merdae	98%	Firmicutes	Clostridia	Clostridiales	Clostridiaceae	Clostridium	40% (16)	45.7% (16)
Dialister succinatiphilus	100%	Firmicutes	Negativicutes	Veillonellales	Veillonellaceae	Dialister	52.5% (21)	62.8% (22)
Enterococcus faecium	94%	Firmicutes	Bacilli	Lactobacillales	Enterococcaceae	Enterococcus	32.5% (13)	11.4% (4)
Faecalibacterium prausnitzii	100%	Firmicutes	Clostridia	Clostridiales	Ruminococcaceae	Faecalibacterium	55% (22)	74.3% (26)
Fusicatenibacter saccharivorans	100%	Firmicutes	Clostridia	Clostridiales	Lachnospiraceae	Fusicatenibacter	90% (36)	97.1% (34)
Negativibacillus massiliensis	91%	Firmicutes	Clostridia	Clostridiales	Ruminococcaceae	Negativibacillus	32.5% (13)	48.6% (17)
Prevotellamassilia timonensis	100%	Bacteroidetes	Bacteroidia	Bacteroidales	Prevotellaceae	Prevotellamassilia	30% (12)	40% (14)
Pseudoflavonifractor phocaeensis	96%	Firmicutes	Clostridia	Clostridiales	Pseudoflavonifractor		65% (26)	62.8% (22)
Romboutsia timonensis	100%	Firmicutes	Clostridia	Clostridiales	Peptostreptococcaceae	Romboutsia	35% (14)	14.3% (5)
Subdoligranulum variabile	98%	Firmicutes	Clostridia	Clostridiales	Ruminococcaceae	Subdoligranulum	77.5% (31)	97.1% (34)
Succinivibrio dextrinosolvens	98%	Proteobacteria	Gammaproteobacteria	Aeromonadales	Succinivibrionaceae	Succinivibrio	35% (14)	45.7% (16)
Urmitella timonensis	88%	Firmicutes	Tissierellia	Tissierellales	Tissierellaceae	Urmitella	12.5% (5)	28.6% (10)

Supplementary Figure 2 – DeSeq2 output on the significant different abundance OTUs at onset versus follow-up

Taxonomy	log2FoldChange	padj
f__Ruminococcaceae; g__Ruminococcus; s__bromii	6.8582425583863795	4.07872554853171e-18
f__Ruminococcaceae; g__Ruminococcus; s__bromii	6.228560492417031	9.908434381837149e-18
f__Bacteroidaceae; g__Bacteroides; s__	4.3174375633941695	1.64499055535548e-09
f__Bacteroidaceae; g__Bacteroides; s__	-3.33153593924656	2.80402639094411e-07
f__Rikenellaceae; g__; s__	4.14231024243649	8.9046205265063e-07
f__Lachnospiraceae; g__; s__	3.64441193542444	8.9046205265063e-07
f__Ruminococcaceae; g__; s__	3.5952281825985004	8.9046205265063e-07
f__Succinivibrionaceae; g__Succinivibrio; s__	-2.8126691047417003	3.89157079230104e-06
o__Clostridiales; g__; s__	-3.3558273111461	5.27684538611556e-06
f__Succinivibrionaceae; g__Succinivibrio; s__	-2.5619353989615496	1.8870077018353603e-05
f__Prevotellaceae; g__Prevotella; s__copri	-2.4047117204187503	3.01753918137896e-05
f__Bacteroidaceae; g__Bacteroides; s__	-2.5510986909309796	3.9892661066596703e-05
f__Pasteurellaceae; g__Haemophilus; s__parainfluenzae	-2.21729225366332	9.165223241453241e-05
f__Prevotellaceae; g__Prevotella; s__copri	2.69346887506914	9.78716942388598e-05
f__Prevotellaceae; g__Prevotella; s__copri	2.7437779449382202	9.78716942388598e-05
f__Prevotellaceae; g__Prevotella; s__copri	2.6828315774549103	0.000183344741417528
f__Prevotellaceae; g__Prevotella; s__copri	2.55610614506354	0.000244367449918506
f__[Barnesiellaceae]; g__; s__	-2.11165222864131	0.000251831583080717
f__Ruminococcaceae; g__Ruminococcus; s__	2.53289867439502	0.000260856694348889
f__Ruminococcaceae; g__Ruminococcus; s__	-2.33799283692434	0.000338115771452337
f__Ruminococcaceae; g__Faecalibacterium; s__prausnitzii	-1.9066886740902702	0.000801073050570078
f__Prevotellaceae; g__Prevotella; s__copri	2.82164659333076	0.000872813545438263
